# Nomogram prediction model for prognosis of patients with amyotrophic lateral sclerosis

**DOI:** 10.1186/s12883-026-04741-8

**Published:** 2026-02-26

**Authors:** Qionghua Sun, Hongfen Wang, Guochao Deng, Yuguo Du, Tie Ma, Jichao Ding, Zhenxi Xia, Yuqing Jiang, Yonghua Huang, Xusheng Huang

**Affiliations:** 1 Department of Geriatric Medicine, Seventh Medical Center, Chinese PLA General Hospital, Beijing, 100700 China; 2 Department of Neurology, First Medical Center, Chinese PLA General Hospital, Beijing, 100853 China; 3 Department of Neurology, Seventh Medical Center, Chinese PLA General Hospital, Beijing, 100700 China; 4 Senior Department of Oncology, Chinese PLA General Hospital, Beijing, 100071 China

**Keywords:** Amyotrophic lateral sclerosis, Nomogram model, Prognosis

## Abstract

**Objectives:**

To analyze the factors affecting prognosis of patients with sporadic amyotrophic lateral sclerosis (ALS), to establish a nomogram predictive model.

**Methods:**

A total of 236 patients with sporadic ALS hospitalized in the Department of Neurology of the First Medical Center, Chinese PLA General Hospital, from March 2011 to November 2021 were enrolled in the study. Basic information and clinical and laboratory data of patients were collected, including sex, age at onset, body mass index, disease duration, diagnostic grade, and serum levels of creatine kinase (CK), creatinine (Cr), uric acid (UA), and ferritin. Kaplan-Meier univariate and multivariate Cox proportional hazard regression models were used to analyze the prognostic factors, and a nomogram predictive model was established.

**Results:**

Univariate analysis showed that ferritin, CK, Cr, age at onset, disease duration, and body mass index (BMI) were all correlated with prognosis of ALS. Multivariate analysis showed that ferritin, Cr, disease duration, age at onset, and BMI were the strongest predictors. ROC curve and correction curve analyses verified the accuracy of the nomogram prediction model.

**Conclusions:**

Ferritin, Cr, disease duration, age at onset, and BMI are independent predictors of survival in patients with ALS. Based on these clinical and biological prognostic factors, we established a quantitative model for predicting survival probability, and may assist in the prognostic evaluation of ALS, pending further validation.

## Background

ALS is a progressive neurodegenerative disease, with an average age at onset of 50–60 years. Most patients die within 3 to 5 years after onset, mainly due to respiratory failure. From a clinical perspective, it is important to develop noninvasive methods to determine, simply and quickly, independent predictors of survival and establish accurate and simple prediction models, which will benefit patient health care and risk stratification in clinical trials. At present, late age at onset, bulbar onset, and rapid progression of muscle weakness after onset are considered to be indicators of poor prognosis and shorter survival [1–3]. Age at onset, and rate of disease progression are independent predictors of survival. Some biological markers, such as neurofilament protein, have been shown to be useful for monitoring disease progression and prognosis [4]. In addition, many studies have shown that ferritin levels are elevated in body fluids of patients with ALS, suggesting its potential for predicting prognosis [5, 6]. Its level is related to disease progression, and high ferritin level has been shown to indicate poor prognosis [7]. Another study showed that the levels of ferritin heavy and light chains in cerebrospinal fluid (CSF) can be used to distinguish ALS from other diseases and as indicators of disease progression [8]. A meta-analysis investigating the possible relations between serum ferritin levels and disease susceptibility in patients with ALS, including 1053 ALS cases and 760 healthy controls from the United Kingdom, United States, France, and Japan, showed that serum ferritin concentrations were significantly elevated in patients with ALS compared to healthy controls [9]. Compared with conventional logistic regression models or Cox regression models, nomograms have the advantage that the value of a given clinical or laboratory indicator can be determined graphically while displaying continuous probabilities. Some studies used nomograms to determine the probability of survival in ALS, but they focused on genetic or other factors [10–12].

The purpose of this study is to use a large sample size to establish a predictive model of ALS that combines multiple clinical data and laboratory indicators, and to build a nomogram to evaluate the predictive power of each parameter. It allows clinicians and even patients to make intuitive, clear and simple judgements on prognosis, and formulate individualized treatment strategies based on this. At the same time, the influence and value of ferritin on the prediction model were evaluated through this model.

## Methods

### Patients

In this study, we collected retrospective data from patients hospitalized at the Department of Neurology of the First Medical Center, Chinese PLA General Hospital, between March 2011 and November 2021. A total of 236 patients with sporadic ALS, with complete laboratory data and survival information were selected, diagnosed as clinically definite, clinically probable, clinically probable-laboratory-supported, or clinically possible ALS by experienced neurologists according to the revised El Escorial criteria [13]. Then, based on a training set ratio verification set of approximatively 7: 3, 165 patients were included in the training set and 71 patients were included in the verification set. Patients with diseases that affect laboratory indicators such as ferritin, creatinine (Cr), and creatine kinase (CK) levels, including hematological diseases, tumors, liver and kidney diseases, and family medical history, were excluded from the study [14–18]. This study was approved by the Ethics Committee of General Hospital of China, and all data were anonymized.

### Clinical data

Clinical data of all patients at admission, including sex, age at onset, body mass index (BMI), time from onset to hospitalization (disease duration), and diagnostic grade (Clinically definite/clinically probable/clinically possible ALS), were collected. Each patient’s laboratory index was also recorded, including serum CK, Cr, uric acid (UA), and ferritin levels.

### Follow-up

Follow-up was conducted by telephone. The date of death or tracheotomy was defined as the end point. Overall survival (OS) was defined as the time from the date of hospitalization until the date of the end point event. Cases in which no endpoint occurred by the end of the study period were censored.

### Nomogram construction

X-tile plots were used to accurately and automatically select the optimum cutoff based on the highest χ² value. Clinical indexes were precisely divided based on the optimal cut-off value generated by X-tile software version 3.6.1 (Yale University School of Medicine, USA). The Kaplan-Meier method and log-rank test were used for univariate survival analysis, meaningful predictive factors were screened out (*P* < 0.05) and Cox’s proportional hazards regression model was used for multivariate analysis. Nomograms were constructed using independent prognostic factors in the training set data. The construction of nomograms for 1-, 2-, and 3-year OS prediction was performed using the RMS software package in R software version 4.3.3 (https://www.r-project.org/). Survival analyses were conducted with the *survival*,* survminer*, and *rms* packages. Time-dependent ROC curve analysis and decision curve analysis were performed using the *timeROC* and *rmda* packages, respectively.

### Validation of nomograms and statistical analysis

Time-dependent ROC curves were generated using the *timeROC* package in R with the nearest neighbor estimation method to account for censored survival data. We used the concordance index (C-index) to measure differences between performance and predicted results of the nomograms. The value of C-index ranged from 0.5 to 1, in which 0.5 considered no discrimination at all and 1.0 represented perfect discrimination. Calibration curves were used to compare the predicted results of the nomogram with the actual results, while the 45-degree line was used as the optimal model. The DCA algorithm can be used as a comprehensive method for evaluating prediction models. All statistical analyses were performed using SPSS software (version 23.0; IBM Corp., Armonk, NY, USA). For continuous variables, the Kolmogorov-Smirnov test was used to determine whether the data had a normal distribution. Continuous normally distributed values are expressed as the mean ± standard deviation and non-normally distributed continuous numerical variables are expressed as quartiles. *P* < 0.05 was considered to indicate a statistically significant difference. In addition, the hazard ratio (HR) and its 95% confidence interval (CI) were also calculated.

## Results

### Demographic characteristics

The demographic factors of 236 sALS patients between March 2011 and November 2021 are shown in Table 1. The training data sets consisted of 165 sALS patients with a mean age at onset was 54.91 ± 10.27 years. Among them, 96 (58.2%) patients were male and 69 (41.8%) were female. The average time from symptom onset to hospitalization was 12 (7.38–22.50) months. Among these patients, 101 were classified as having clinically definite ALS, 42 had clinically probable ALS, and 22 had clinically possible ALS. The validation cohort consisted of 71 sALS patients with a mean age at onset was 54.31 ± 9.31 years. Among them, 44 (62.0%) patients were male and 27 (38.0%) were female. The average time from symptom onset to hospitalization was 12 (8–20) months. Among these patients, 42 were classified as having clinically definite ALS, 20 had clinically probable ALS, and 9 had clinically possible ALS. By November 2021, excluding patients who could not be contacted or were lost to follow-up and those with incomplete clinical data, complete survival data were obtained for 236 patients, of whom 193 had died or received tracheostomy and 43 had survived by the end of the study period. In the training cohort, the 1-, 2-, and 3-year survival times were 69.1%, 43.6%, and 24.1%, respectively. In the validation cohort, the 1-, 2-, and 3-year survival times were 73.2%, 43.6%, and 26.3%, respectively (Table 1).

**Table 1:** Demographic characteristics in the training and validation cohorts

Characteristic	Training cohort (*n* = 165)	Validation cohort (*n* = 71)
	Mean ± SD/No (%)	Mean ± SD/No (%)
Sex		
Male	96 (58.2%)	44 (62.0%)
Female	69 (41.8%)	27 (38.0%)
Onset age (years)	54.91 ± 10.27	54.31 ± 9.31
Disease duration (months)	12 (7.38–22.50)	12 (8–20)
Diagnostic grade		
Clinically definite	101 (61.2%)	42 (59.1%)
Clinically probable	42 (25.5%)	20 (28.2%)
Clinically possible	22 (13.3%)	9 (12.7%)
BMI	23.36 ± 3.40	23.71± 3.16
Survival status		
Death (*n*)	136 (82.4%)	57 (80.3%)
Survive (*n*)	29 (17.6%)	14 (19.7%)
Median survival time (months)	22 (19.365–24.635)	20 (14.259–25.741)
Laboratory indicators		
CK (U/L)	203.97 ± 162.87	184.21 ± 155.86
Cr (µmol/L)	58.81 ± 13.15	59.26 ± 13.77
UA (µmol/L)	297.27 ± 84.50	289.87 ± 67.58
Ferritin (µg/L)	246.69 ± 176.12	213.18 ± 160.52

*BMI* body mass index, *CK* creatine kinase, *Cr* creatinine, *UA* uric acid

### Sifted independent risk factors and development of the nomogram model

**Fig. 1:**
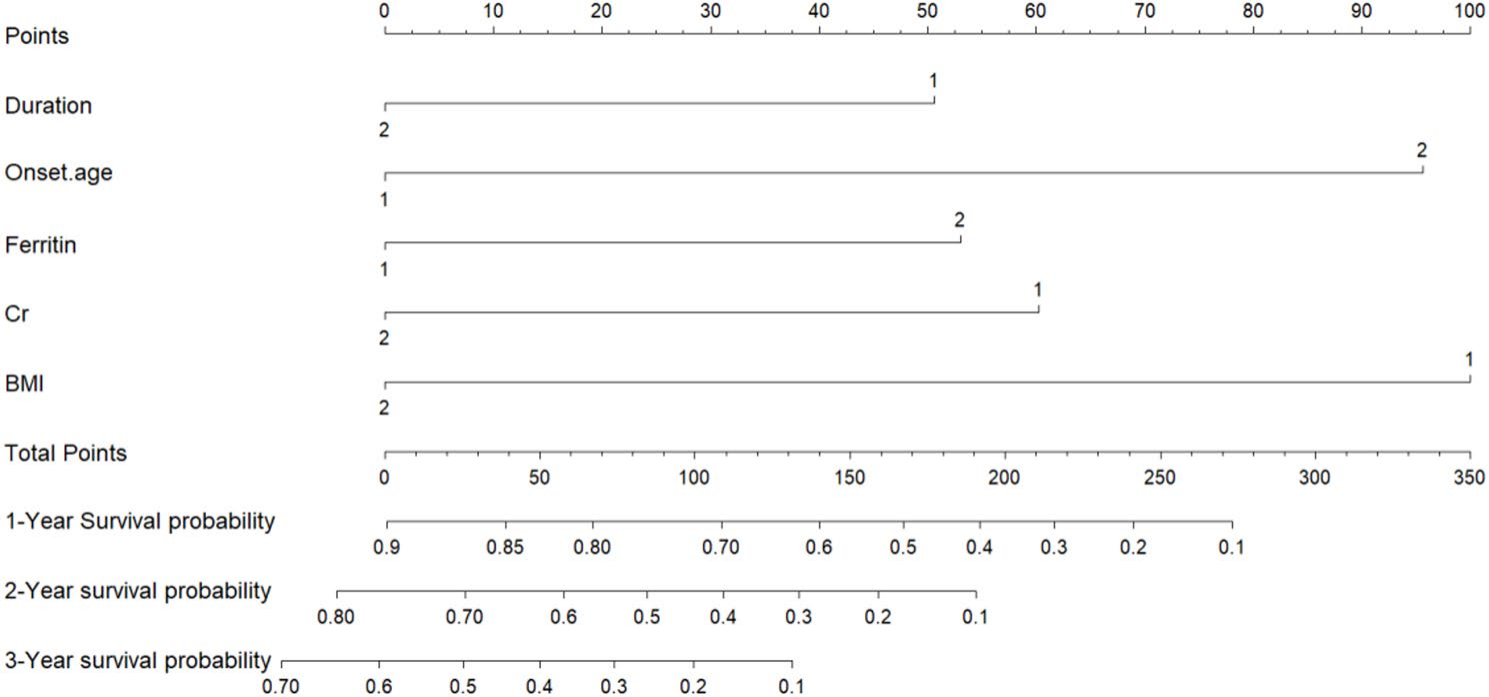
Prognostic nomogram for predicting 1-, 2-, and 3-year overall survival in patients with sporadic ALS. Covariates were assessed for each patient and assigned a point value in the nomogram. A higher total number of points indicates a higher likelihood of poor clinical outcomes and shorter expected survival. Cr: creatinine; BMI: body mass index

Survival time was taken as a dependent variable, with sex, age at onset, diagnostic grade, BMI, disease duration (i.e., time from onset of symptoms to admission), and serum CK, Cr, UA, and ferritin levels were taken as independent variables. The influence of each variable on survival time was examined by the univariate KaplanMeier method and log-rank test. The results showed that CK, Cr, ferritin, age at onset, disease duration, and BMI were related to prognosis of ALS (*P* < 0.1). Sex, diagnostic grade, and UA were not significantly related to prognosis of ALS (*P* > 0.1). These meaningful indicators were incorporated into a Cox risk ratio model. The results showed Cr, ferritin, disease duration, onset age, and BMI were identified as independent predictors of OS (Table 2). Based on the aforementioned results, we combined these 5 predictors (Fig. 1). By projecting the points corresponding to each variable to the “Points” axis, summing the total scores gives the corresponding prediction results.

**Table 2:** Univariate and multivariate Cox proportional hazard analyses of OS with sALS patients

Variable	Univariate analysis		Multivariate analysis	
	HR (95% CI)	*P*-value	HR (95% CI)	*P*-value
Sex (male/female)	0.987 (0.739–1.318)	0.928		
Age at onset	**1.034 (1.018–1.050)**	**< 0.001**	**1.028 (1.012–1.045)**	**0.001**
Disease duration	**0.985 (0.974–0.997)**	**0.012**	**0.980 (0.967–0.993)**	**0.004**
Diagnostic grade	0.873 (0.709–1.074)	0.130		
BMI	**0.945 (0.902–0.990)**	**0.017**	**0.944 (0.899–0.991)**	**0.020**
Ferritin	**1.530(1.058–2.212)**	**0.020**	**1.001 (1.000–1.002)**	**0.004**
CK	**0.999 (0.998–1.000)**	**0.077**	0.999 (0.998–1.000)	0.216
Cr	**0.989 (0.978–1.001)**	**0.065**	**0.984 (0.972–0.996)**	**0.011**
UA	0.999 (0.998–1.001)	0.579		

Bold font indicates *p* < 0.05

*BMI* body mass index, *CK* creatine kinase, *Cr* creatinine, *UA* uric acid

### Performance assessment and validation of the nomogram

The predictive accuracy and clinical applicability of the established nomogram were rigorously assessed in terms of discrimination, calibration, and clinical utility using bootstrap resampling with 1000 repetitions.

**Fig. 2:**
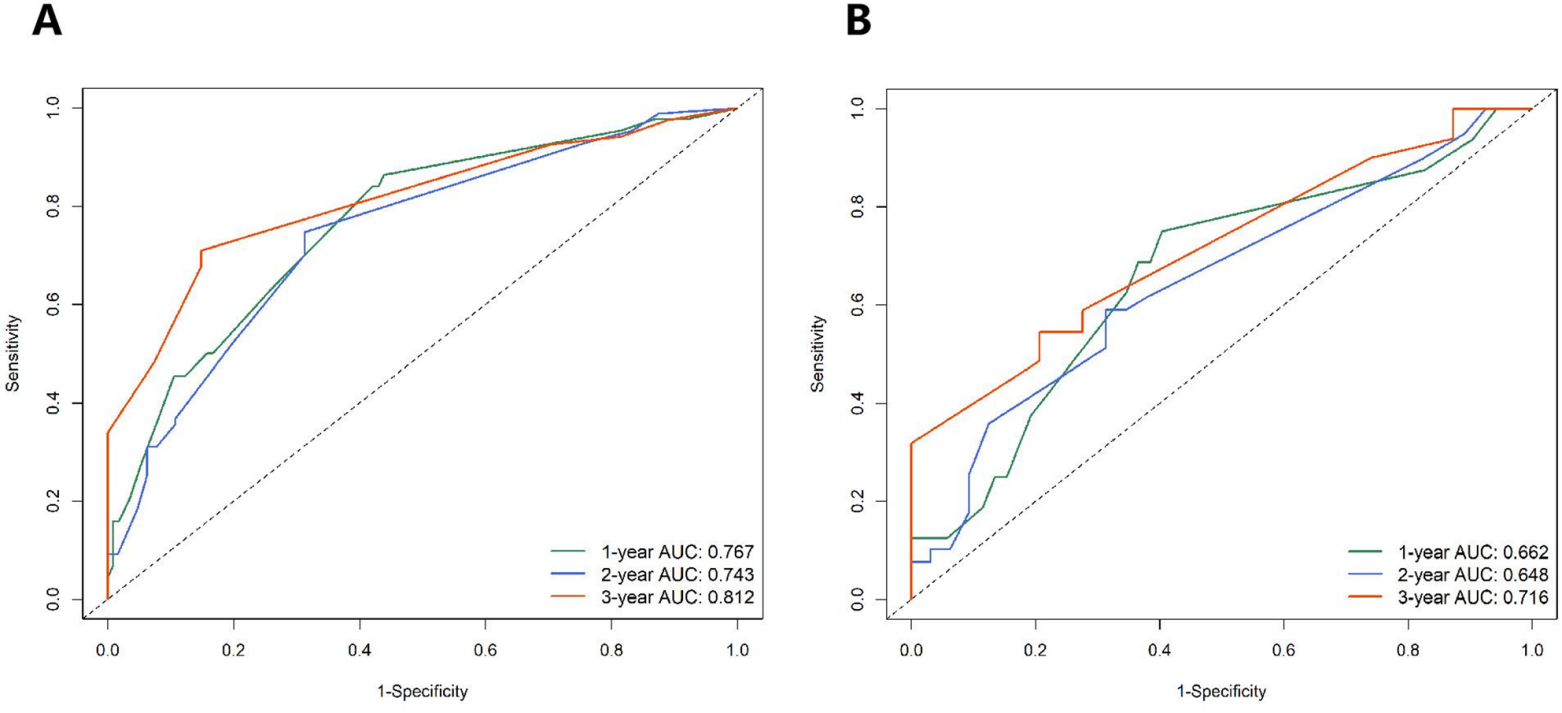
Time-dependent ROC curves for the nomogram model in predicting overall survival at 1, 2, and 3 years in the training (**A**) and validation (**B**) cohorts. The area under the curve (AUC) values are displayed for each time point. AUC values > 0.7 are generally considered indicative of a useful predictive model

Discriminative ability was evaluated using time-dependent receiver operating characteristic (ROC) curve analysis. In the training cohort, the nomogram demonstrated good predictive accuracy for survival, with area under the curve (AUC) values of 0.767, 0.743, and 0.812 for predicting 1-, 2-, and 3-year overall survival, respectively (Fig. 2A). In the independent validation cohort, the model maintained acceptable discriminative performance, with corresponding AUCs of 0.662, 0.648, and 0.716 (Fig. 2B). AUC values above 0.7 are generally considered to indicate a useful model. The training set results notably meet this criterion, while the validation set results, all exceeding 0.65, confirm the model’s satisfactory and stable discriminatory ability. The model demonstrated good discriminative ability, with a C-index of 0.699 (95% CI: 0.650–0.747) in the training cohort and 0.626 (95% CI: 0.548–0.704) in the validation cohort.

**Fig. 3:**
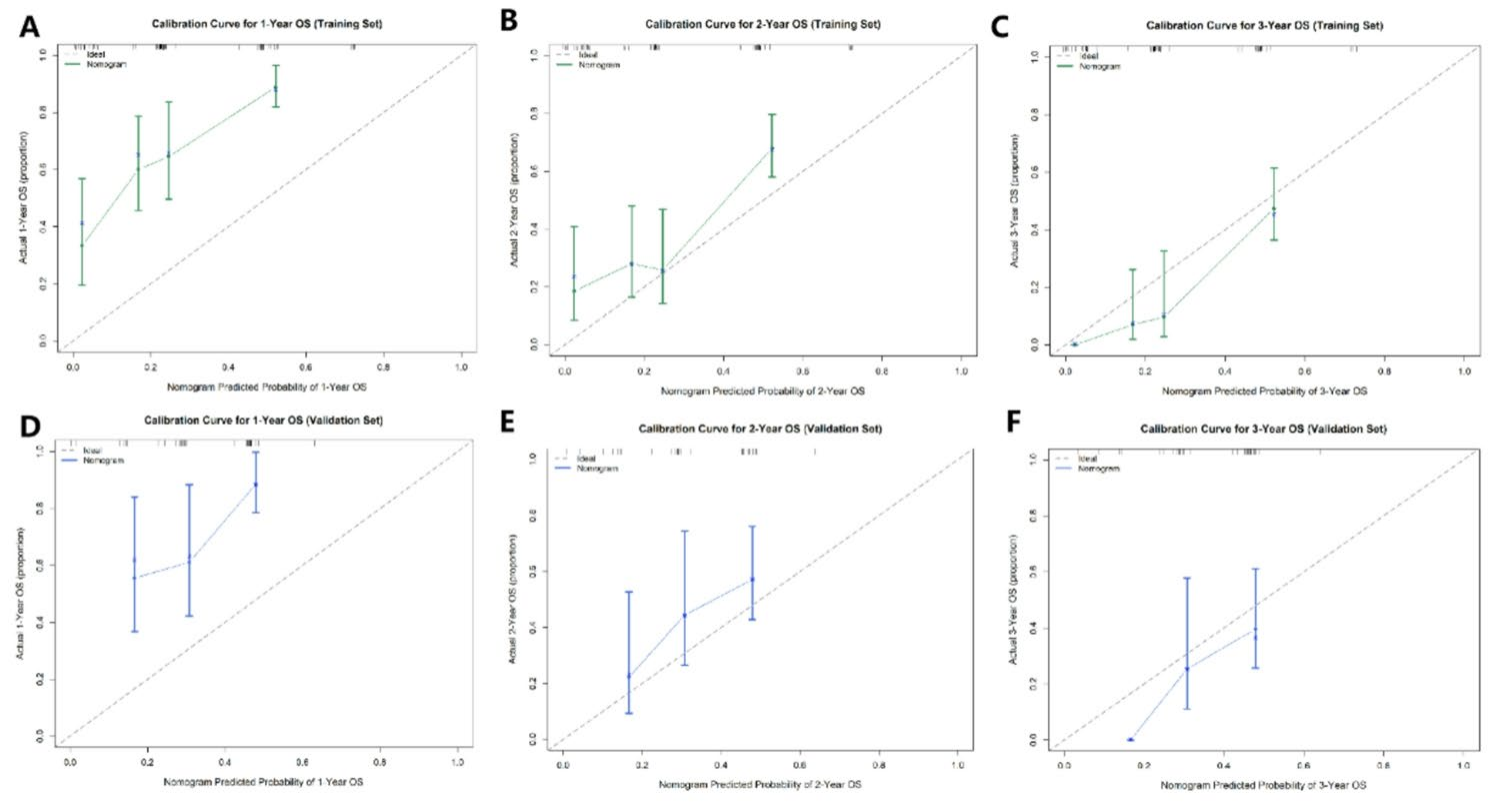
Calibration curves of the nomogram for predicting 1-, 2-, and 3-year overall survival in the training (**A**–**C**) and validation (**D**–**F**) cohorts. The solid lines represent the observed survival probabilities predicted by the nomogram, and the dashed 45-degree lines represent the ideal perfect prediction. **Calibration curves were optimism-corrected via bootstrap resampling (1000 repetitions). **close alignment indicates good calibration accuracy

Calibration, which evaluates the agreement between predicted probabilities and actual observed outcomes, was visualized using optimism-corrected calibration curves generated via bootstrap resampling (1000 repetitions). As shown in Fig. 3, the solid lines representing our nomogram’s predictions closely followed the 45-degree dashed ideal line across both the training (Fig. 3A-C) and validation (Fig. 3D-F) sets for 1-, 2-, and 3-year survival. This close alignment demonstrates excellent calibration, meaning the nomogram’s predicted survival probabilities are highly accurate and reliable in both cohorts.

**Fig. 4:**
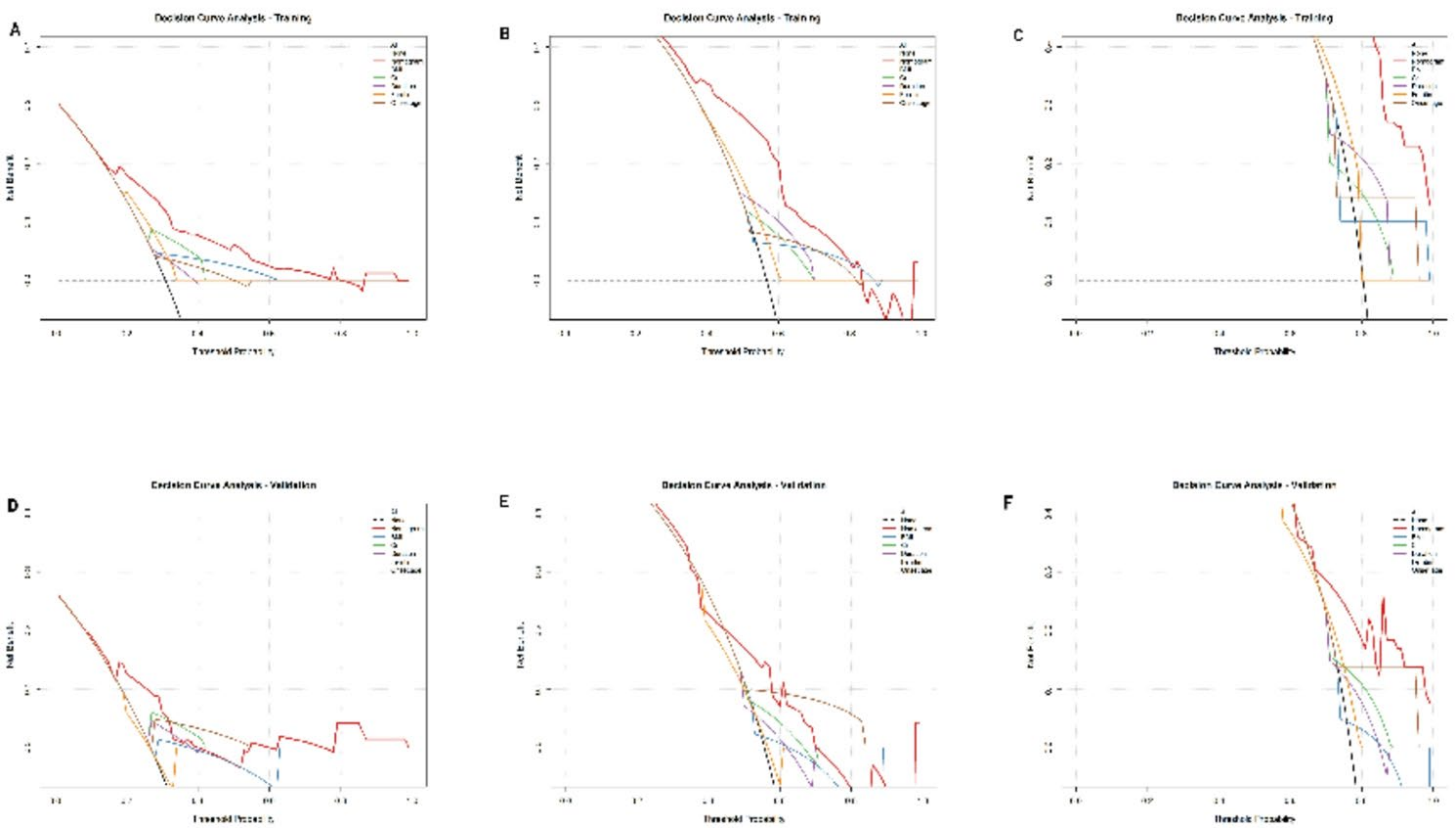
Decision curve analysis (DCA) for the nomogram predicting 1-, 2-, and 3-year overall survival in the training (**A**–**C**) and validation (**D**–**F**) cohorts. The red lines depict the net benefit of using the nomogram for clinical decision-making. The green and black lines represent the net benefit of the strategies of “treating all patients” and “treating no patients,” respectively. The nomogram shows superior net benefit across a range of threshold probabilities. BMI, body mass index; Cr: creatinine

To evaluate the clinical net benefit and utility of applying the nomogram in decision-making, we performed Decision Curve Analysis (DCA). The DCA curves for 1-, 2-, and 3-year survival in both the training and validation sets are presented in Fig. 4. Across a wide range of clinically reasonable threshold probabilities, the nomogram (red line) consistently yielded a higher net benefit than the strategies of “treating all patients” (green line) or “treating no patients” (black line). Furthermore, the nomogram’s net benefit surpassed that of any single predictor variable (BMI, Cr, Duration, Ferritin, Onset age), underscoring its superior clinical value as a comprehensive tool. This indicates that using our nomogram to guide clinical decisions would lead to better patient outcomes with fewer unnecessary interventions compared to relying on individual factors or simple rules.

In summary, the validation results demonstrate that our nomogram possesses strong discriminatory power, high calibration accuracy, and a favorable clinical net benefit. This suggests that our nomogram demonstrates promising predictive performance and may serve as a helpful non-invasive, quantitative tool for individualized survival prediction in patients with sporadic ALS at the time of hospital admission.

## Discussion

Most previous studies used Cox proportional hazards analysis or Kaplan-Meier curves to determine predictors of survival time in patients with ALS [19–21]. A few studies used logistic regression analysis to assess the associations between clinical markers and survival time in patients with ALS [22]. Nomogram prediction models have been developed for some cancers and have been shown to predict prognosis more accurately than traditional staging systems. The prognostic nomogram constructed here included independent risk factors related to survival in patients with ALS, which was verified by ROC curves and correction curves. The results showed that the model was accurate and reliable for estimating the survival of patients with ALS. This map provided an effective method for predicting the likely survival of patients with ALS, and can be used to provide more prognostic information for some patients with ALS. This model map will be useful for patients with ALS to obtain quantitative information about disease progression and will help clinicians to arrange appropriate treatment and management. These findings will also be important for the design of future clinical trials. During ALS disease progression, many factors are thought to be closely related to prognosis and have been verified by many large sample studies, including time of onset, delay in diagnosis, etc [1, 23]. In this study, we incorporated these clinical parameters into the model. In addition, among biological markers, CK and Cr can reflect muscle metabolism and are related to progression of muscle weakness and degree of muscle atrophy [19, 24]. BMI can reflect the change in a patient’s total muscle volume [25]. Rapid muscle atrophy and progressive emaciation are often associated with poor prognosis. Multivariate analysis and the nomogram model showed that age at onset, Cr, ferritin, BMI, and time from onset to hospitalization were independent predictors of survival time, while other factors, including sex, location of disease, diagnosis level at admission, and CK and UA levels at admission, were not predictive.

In this study, we constructed a nomogram model to predict the prognosis of ALS based on five independent risk factors: age at onset, Cr, BMI, duration of disease, and ferritin level. The model showed good discrimination and consistency, and provided both explanatory and clinical evidence for prognostic assessment in patients with ALS. The predictive value of ferritin level for survival time was further quantified, enabling individualized predictions at the time of hospitalization. Understanding survival probability based on ferritin levels can help clinicians provide more evidence-based attention and adopt more appropriate treatment strategies.

Several limitations of this study should be acknowledged. First, its retrospective, single-center design may limit the generalizability of our findings and introduce potential selection bias. Future validation through prospective, multi-center studies is warranted. Second, our model did not incorporate several established prognostic factors, such as the ALS Functional Rating Scale-Revised (ALSFRS-R) score and respiratory function parameters (e.g., forced vital capacity), which are known to correlate strongly with disease progression. Third, and importantly, data on disease-modifying treatments (e.g., riluzole, edaravone) and supportive interventions (e.g., non-invasive ventilation, percutaneous endoscopic gastrostomy) were not systematically collected. The use of these therapies can significantly influence survival and represents a potential confounding factor.

The single-center, retrospective design and the absence of data on ALSFRS-R, respiratory function, and disease-modifying treatments are important limitations that affect generalizability. Future studies should aim to include comprehensive treatment information to develop more robust and clinically applicable prognostic tools.

## Conclusions

Ferritin level, Cr level, disease duration, age at onset, and BMI are independent predictors of survival in patients with ALS. Based on these clinical and biological prognostic factors, we established a quantitative model that provides an effective method for estimating patient survival probability and offers a preliminary basis for accurate clinical assessment of ALS prognosis, thereby supporting the development of personalized treatment strategies.

## Data Availability

All data generated or analyzed during this study are included in the article. Further enquiries can be directed to the corresponding author.
